# Association between ceramides and coronary artery stenosis in patients with coronary artery disease

**DOI:** 10.1186/s12944-020-01329-0

**Published:** 2020-06-25

**Authors:** Chenchen Tu, Lan Xie, Zhenjie Wang, Lili Zhang, Hongmei Wu, Wei Ni, Caixia Li, Lin Li, Yong Zeng

**Affiliations:** 1 Department of Cardiology, Beijing Anzhen Hospital, Capital Medical University, No. 2 Anzhen Rd, Chaoyang District, Beijing, 100029 China; 2 Beijing Health Biotech Co. Ltd., yard 7, science park road, Huilongguan, Changping district, Beijing, 102206 China; 3 Health Examination Center, Peking Union Medical College Hospital, No. 41 Damucang Hutong, Xicheng District, Beijing, 100032 China

**Keywords:** Ceramide, Coronary stenosis, Acute coronary syndrome, Acute myocardial infarction, Major adverse cardiac and cerebrovascular events, Liquid chromatography-tandem mass spectrometry

## Abstract

**Background:**

Coronary artery stenosis induces heart diseases including acute coronary syndrome (ACS). Some studies reported the ceramide species are associated with the ACS and major adverse cardia and cerebrovascular events (MACE). However, few studies investigated the association between plasma ceramide levels and the severity of stenosis, together with the onset of diseases. This aim of the present study was to investigate the association betweencertain ceramide species, coronary artery stenosis and acute coronary syndrome.

**Methods:**

Five hundred fifty-three patients with definite or suspected CAD were recruited and received angiography. Subjects were assigned into 4 groups according to the severity of coronary artery stenosis. The measurements of 4 plasma ceramide species, namely, Cer (d18:1/16:0), Cer (d18:1/18:0), Cer (d18:1/24:1), Cer (d18:1/24:0) were carried out by Liquid chromatography-tandem mass spectrometry (LC-MS/MS) and the ratio of Cer (d18:1/16:0), Cer (d18:1/18:0) and Cer (d18:1/24:1) to Cer (18:1/24:0), respectively, were calculated as index to evaluate the association between plasma ceramides levels and coronary artery stenosis. Multiple logistic regression analysis was used to establish the prognostic model for the prediction of ACS risk.

**Results:**

After the adjustment by multiple clinical risk factors including age, gender, pre-existing myocardial/cerebral infarction, hemoglobin A1c% (HbA1c%), smoking and the diagnosis during index hospitalization, multiple logistic regression analysis showed that the high ratio of Cer (d18:1/24:1) to Cer (d18:1/24:0), female gender, HbA1c%, unstable angina (UAP) and acute myocardial infarction (AMI) diagnosis (compared with atherosclerosis) during index hospitalization were associated with more severe coronary artery stenosis. Furthermore, the prognostic model was established after adjustment of risk factors and the area under curve (AUC) of receiver operating characteristics (ROC) for the prognostic model was 0.732 and 95% CI was 0.642–0.822.

**Conclusion:**

The severity of coronary artery stenosis is associated with high ratio of Cer (d18:1/24:1) to Cer (d18:1/24:0), female gender, HbA1c% and AMI. Although the reported prognostic model showed a good discrimination, further investigation on long term MACE is needed to evaluate the role of ceramide for the prediction of MACE risk.

## Background

Acute coronary syndrome (ACS) refers to a spectrum of clinical presentations ranging from acute myocardial infarction (AMI) including ST-segment elevation myocardial infarction (STEMI) and non-ST-segment elevation myocardial infarction (NSTEMI) to presentations found in unstable angina (UAP). The high mortality of ACS is associated with rupture of an atherosclerotic plaque and partial or complete thrombosis of the infarct-related artery [1, 2]. Atherosclerosis results from the buildup of lipids, macrophages, T-lymphocytes, smooth muscle cells, extracellular matrix, calcium and necrotic debris in vessels. Therefore, the lipids (Low Density Lipoprotein (LDL), high-density lipoprotein (HDL), and triglyceride) and inflammation factors (cysteine protease inhibitor C and high-sensitivity C-reactive protein (hs-CRP)) combined with biomarkers of cardiac disease (brain natriuretic peptide (BNP), creatine kinase-MB (CK-MB) and cardiac troponin I (cTnl) etc.) are widely used in clinic diagnosis of ACS and evaluation of ACS risk.

Ceramide is a family of sphingomeric lipids composed of long chain bases of sphingosine and fatty acids heads. In the past few years, numerous studies have suggested that ceramides and other sphingolipids regulate cellular responses to extracellular stimuli and stress and they are involved in pathophysiological mechanism in many disease areas [3–6]. It has been proposed and confirmed by several researchers [7, 8] that plasma ceramide level increased drastically in the presence of high level of LDL cholesterol. Plasma ceramide levelis also positively associated with the levels of total cholesterol. More recent clinical studies [9–14] worldwide have rasied the theory that some specific plasma ceramides can be used as biomarkers, which are more precise than traditional lipid biomarkers for the prediction of adverse cardiovascular outcomes in patients with coronary artery disease (CAD) or the risk of acute coronary syndrome in healthy cohort. The plasma ceramides that have been studied as high-risk factors are Cer (d18:1/16:0), Cer (d18:1/18:0) and Cer (d18:1/24:1) and the ratios of these ceramides to Cer (d18:1/24:0) [15, 16]. Although these studies suggest that plasma ceramide may contribute to atherogenesis and correlate with the risk of coronary heart disease, the solid clinical evidence indicating the association between the plasma ceramides and the coronary artery stenosis and the incidence of ACS is still inadequate.

The present study investigated the association between plasma ceramide species, coronary artery stenosis and ACS in patients undergoing coronary artery angiography with established ACS and other CAD.

## Method

### Subject and sample

Five hundred fifty-three patients with suspected or definite ACS and other CAD who underwent angiography in Beijing Anzhen hospital between Mar.2018 and Aug.2018 were recruited. The exclusion criteria included moderate to severe chronic kidney disease, suspected aortic dissection, acute pulmonary embolism, familial hypercholesterolemia, drug abuse, alcohol dependence, history of percutaneous coronary intervention (PCI) and coronary artery bypass grafting (CABG), and history of stroke in the last 3 months before recruiting. In addition, the patients who needed emergency PCI were excluded in this study including patients were STEMI occurred within 24 h, non-STEMI with symptoms like chest pain and unstable hemodynamics. The diagnostic criteria were adopted from WHO criteria and ESC Guidelines [17–19]. Briefly, the criteria included electrocardiograph (ECG) and the presence or absence of serologic markers. ST elevations, ST depressions, T-wave inversions and pathological Q-waves may be observed both in AMI and UAP patients, but they were transient in UAP patients. Cardiac troponins elevation in peripheral blood was for the establishment of a diagnosis of AMI, whereas cardiac troponins may slightly increase in UAP but did not meet criteria for AMI. CK-MB was elevated in AMI but not in UAP. Both normal ECG and normal levels of cardiac troponins and CK-MB were presented in atherosclerosis.

All patients fasted overnight before the scheduled day for angiography. About 500 μL of plasma samples were collected from each participated patient in the morning before the coronary angiography surgery and stored at − 80 °C until analysis.

**Fig. 1:**
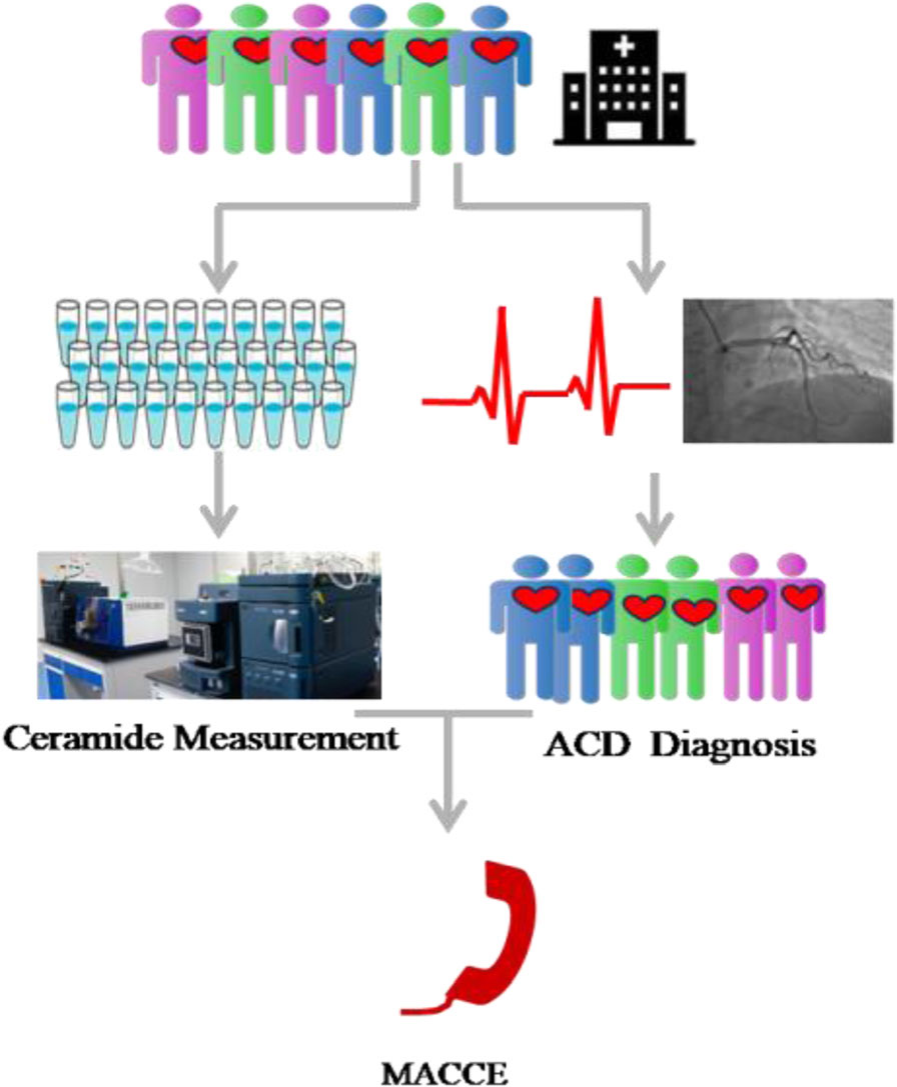
Work flow of the study

The clinic records included age, gender, history of cardiac and cerebrovascular infarction, smoking, alcohol assumption, history of medication and blood chemistry tests. The coronary stenosis severity was grated using semi-quantitative stenosis grading system based on angiographic images. The patients were contacted by phone up to 1 year after discharging to ascertain the incidence of MACE, which included myocardial infarction, stent thrombosis, target vessel revascularization, stroke and cardiac death. The workflow is presented in Fig. 1.

The Ethics Committee of Anzhen Hospital approved the study protocol with the approval number 2017060X. All participants submitted their written informed consent for the participation in this research.

### Angiography and grouping

Before coronary angiography, the arm or groin of patient was cleaned and numbed with 1% lidocaine, followed by puncturing the radial artery or the femoral artery. Then a catheter was put through the artery and carefully moved up into the coronary artery. Once the catheter was in place, contrast material (Iopromide, Bayer Schering Pharma AG, Berlin, Germany) was injected into the catheter. X-ray images were taken at different angles at the same time. The obtained angiographic films were assessed by experienced clinical cardiologist to get semi-quantify coronary artery stenosis.

**Fig. 2:**
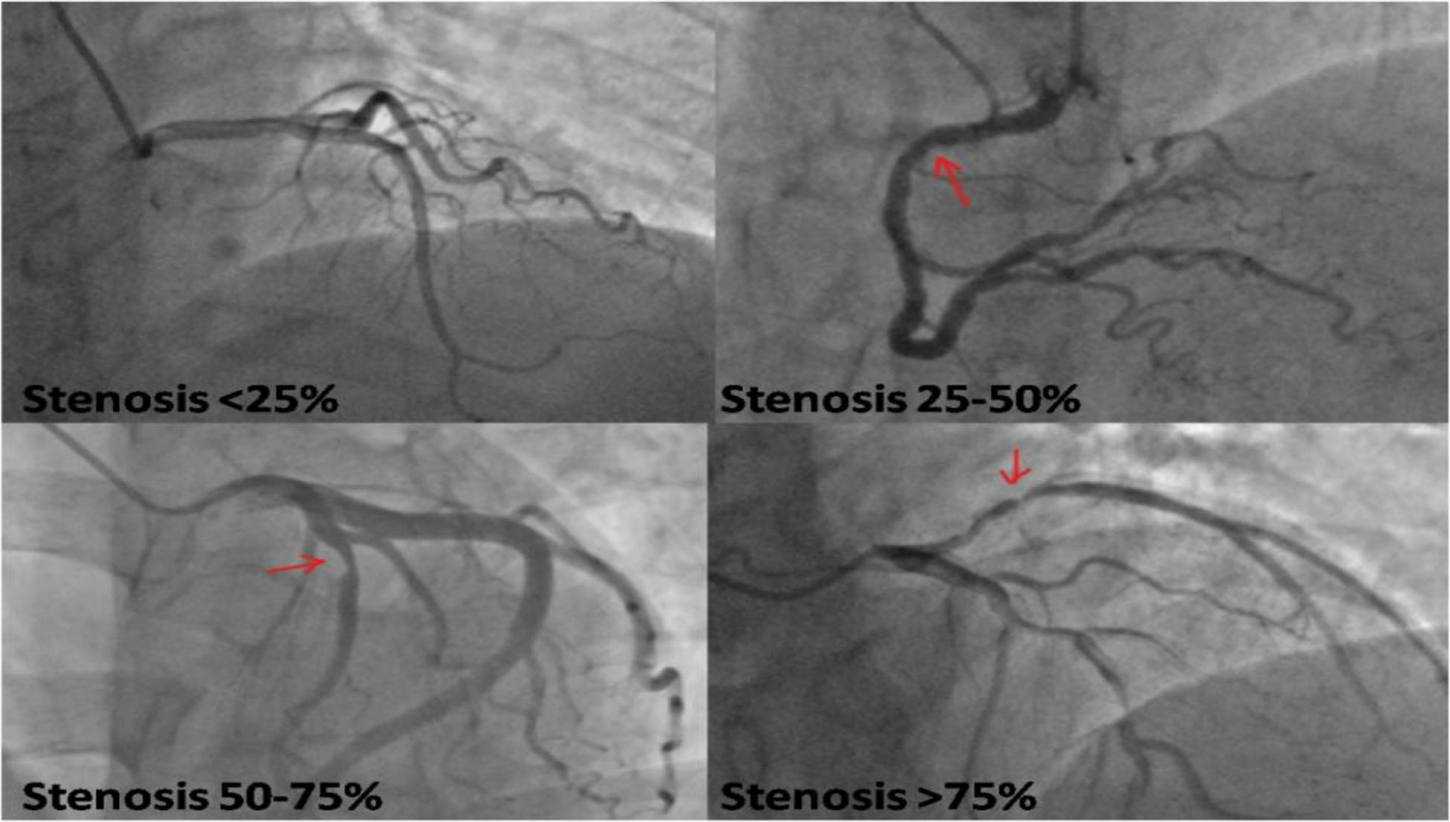
The representative image of coronary artery stenosis

The largest percentage of coronary artery stenosis in single vessel (left main artery, left anterior descending, right coronary artery or left circumflex artery) defined the severity of coronary artery stenosis. In all subjects, 93 out of 107 subjects with stenosis < 50% were single vessel CAD, while 10 and 4 subjects were 2 vessels and 3 vessels CAD, respectively. Most multi-vessel CAD patients (96.4%) were in group of stenosis > 50%, and only 2 out of 368 cases were single vessel CAD with stenosis > 75%. Therefore, the patients were assigned into 4 groups according to the severity of coronary artery stenosis: group 1: stenosis < 25% (*n* = 60); group 2: stenosis 25–50% (*n* = 47); group 3: stenosis 50–75% (*n* = 78); group 4: stenosis > 75% (*n* = 368). The representative angiographic images are shown in picture (Fig. 2).

### Ceramide measurement

The LC-MS/MS conditions, method performance, and evaluation of the methodology are described in supplementary information (Additional file 1).

### Statistical analysis

One way ANOVA followed by post-hoc test and Chi-square test were used to analyze the differences in main clinical and biochemical characteristics of patients. Kruskal-Wallis test and Mann-Whitney test were used for ceramide levels analysis. Multiple logistic stepwise regression analysis was used to evaluate the independent association between coronary artery stenosis and risk factors including plasma ceramide species. The prognostic association of individual ceramide species with MACE was modelled using logistic regression.

## Result

### Subject profile

A total of 553 patients with a primary diagnosis as coronary heart disease and underwent the coronary angiography were recruited. The clinical characteristics and part of chemistry profiles of the subjects are shown in table (Table 1).

**Table 1:** Clinical and Biochemistry characteristics of Patients

Characteristic	Coronary Stenosis				*P*
	< 25%, ***N*** = 60	25–50%, ***N*** = 47	50–75%, ***N*** = 78	>75%, ***N*** =368	
Age, yrs	56 ± 10	61 ± 9	59 ± 10	60 ± 11	0.055
Female	35 (58.3%)	11 (23.4%)	15 (19.2%)	94(25.6%)	< 0.001
BMI, kg/m2	25.7 ± 5.1	25.3 ± 2.6	26.8 ± 6.4	25.4 ± 5.2	0.302
Smoking	19 (31.7%)	25 (52.1%)	48(61.5%)	198 (53.8%)	0.02
Diabetes	13 (21.7%)	15 (31.3%)	28 (35.9%)	123 (33.4%)	0.224
hypertension	31 (51.7%)	28(58.3%)	46(60.0%)	255 (69.3%)	0.021
Prior MI/CI	3 (5.0%)	6 (12.5%)	11 (14.1%)	56 (15.2%)	0.183
Total cholesterol, mmol/L	3.997 ± 0.894	3.859 ± 0.945	3.971 ± 0.946	3.960 ± 1.056	0.890
HDL-C, mmol/L	1.157 ± 0.250	1.120 ± 0.285	1.045 ± 0.206	1.037 ± 0.261	0.004
LDL-C, mmol/L	2.281 ± 0.682	2.139 ± 0.724	2.401 ± 0.788	2.328 ± 0.800	0.286
HbA1c%	6.027 ± 1.071	6.320 ± 0.914	6.394 ± 1.099	6.598 ± 1.176	0.002
Cer (d18:1/16:0), μmol/L	0.226 ± 0.086	0.202 ± 0.075	0.217 ± 0.673	0.223 ± 0.085	0.371
Cer (d18:1/18:0), μmol/L	0.070 ± 0.043	0.057 ± 0.022	0.068 ± 0.030	0.068 ± 0.035	0.280
Cer (d18:1/24:1), μmol/L	2.463 ± 1.007	2.587 ± 0.996	2.620 ± 0.996	2.551 ± 0.993	0.756
Cer (d18:1/24:0), μmol/L	0.746 ± 0.395	0.694 ± 0.241	0.776 ± 0.294	0.782 ± 0.336	0.294
Cer (d18:1/16:0)/Cer (18:1/24:0)	0.096 ± 0.031	0.083 ± 0.026	0.088 ± 0.025	0.092 ± 0.030	0.049
Cer (d18:1/18:0)/Cer (18:1/24:0)	0.029 ± 0.015	0.024 ± 0.011	0.028 ± 0.013	0.028 ± 0.012	0.114
Cer (d18:1/24:1)/Cer (18:1/24:0)	0.322 ± 0.161	0.290 ± 0.130	0.318 ± 0.135	0.329 ± 0.148	0.108
hsCRP, mg/L	0.9(1.37)	0.95(1.507)	1.0(2.085)	1.05(2.32)	0.095
eGFR, mL/min/1.73m^2^	99.438 ± 14.123	97.974 ± 10.433	94.436 ± 17.180	92.792 ± 18.086	0.003
GRACE score	108.330 ± 23.281	105.945 ± 17.739	105.932 ± 22.645	105.736 ± 26.822	0.381
current medications (statins)	54(90.0%)	46(97.9%)	75(96.2%)	361(98.1%)	0.009

The clinical characteristics in coronary artery stenosis patients. (data is presented as mean ± SD, median and IQR or percentage)

### Plasma ceramide level in ACS and atherosclerosis

**Fig. 3:**
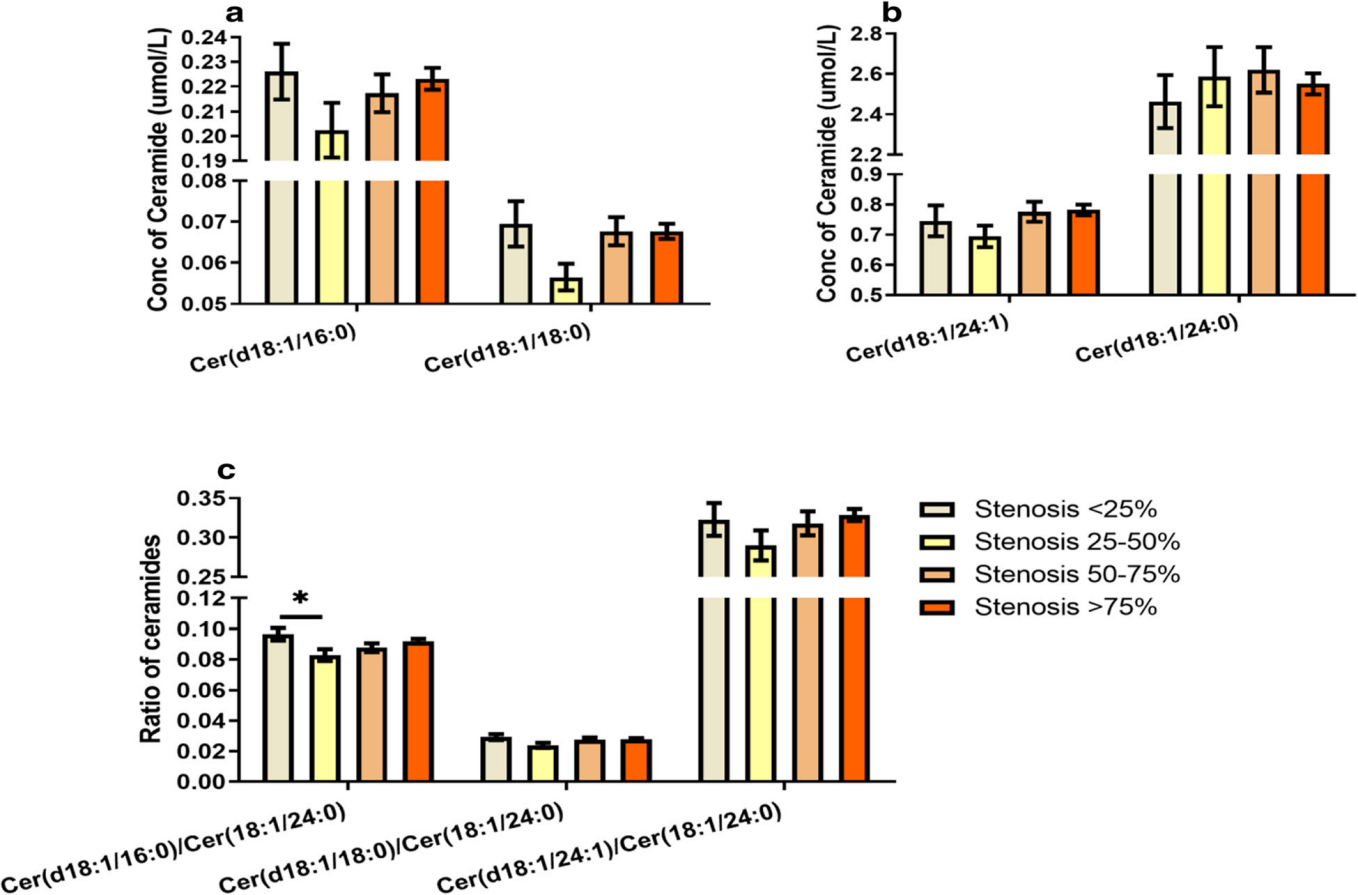
Concentration of ceramides and ratio of ceramides to cer (d18:1/24:0). **a.** concentration of cer (d18:1/16:0) and cer (d18:1/18:0): there were no significant differences in cer (d18:1/16:0) or cer (d18:1/18:0) level between groups. **b.** concentration of cer (d18:1/24:1) and cer (d18:1/24:0): neither cer (d18:1/24:1) nor cer (d18:1/24:0) were significantly different between groups. **c.** ratio of ceramides to cer (d18:1/24:0): ratio of cer (d18:1/16:0) to cer (d18:1/24:0) was significantly increased in group with stenosis < 25% compared to group with stenosis 25–50% (*P* < 0.05), whereas there was no difference between other groups. There were no significant differences in ratios of cer (d18:1/18:0) or cer (d18:1/24:1) to cer (d18:1/24:0) between groups. (data is presented as mean ± SEM)

**Fig. 4:**
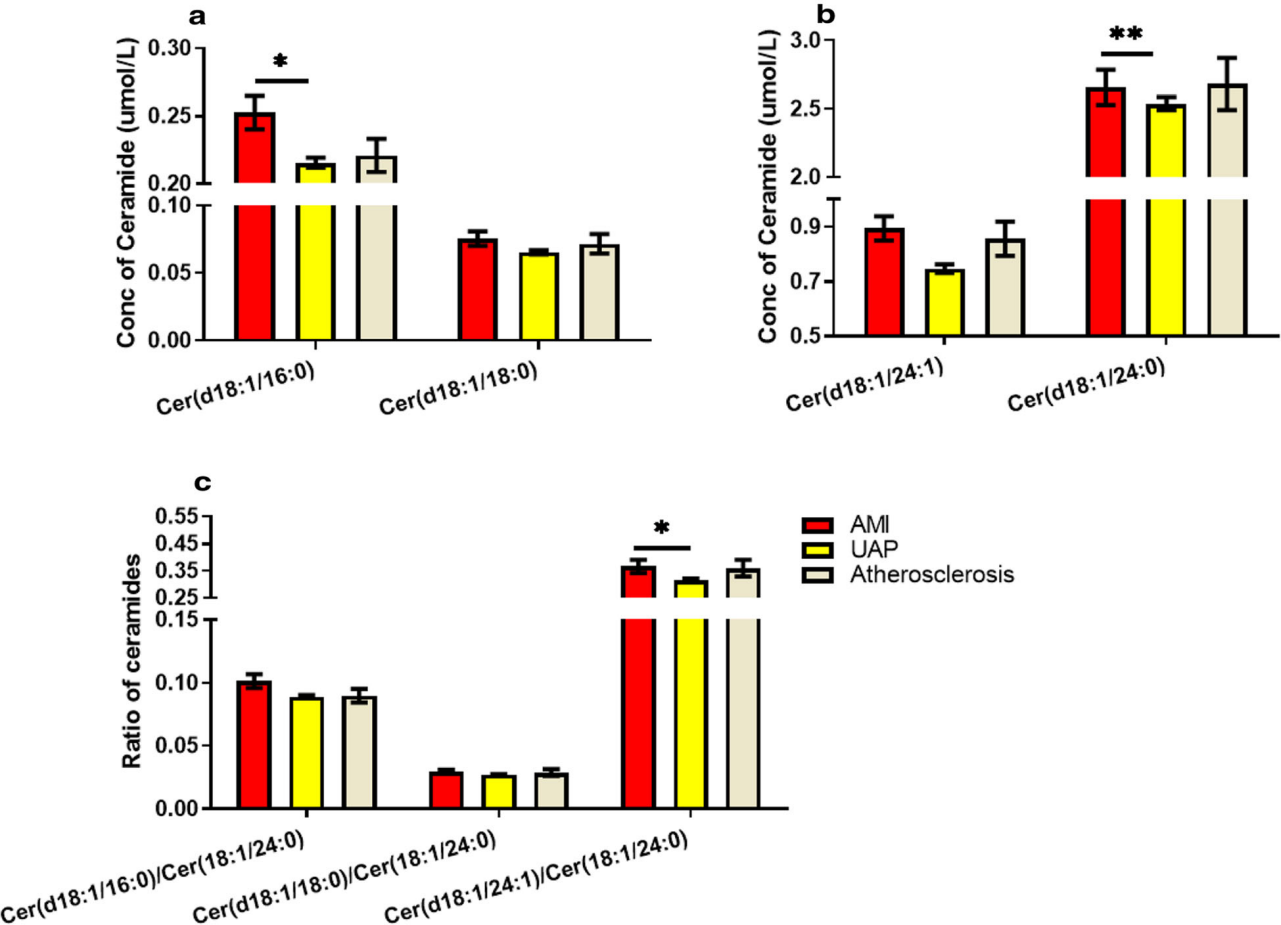
Level of plasma ceramide species in ACS and atherosclerosis patient: **a.** concentration of cer (d18:1/16:0) and cer (d18:1/18:0): cer (d18:1/16:0) concentration wasonly significantly elevated in AMI compared to those in UAP (*P* < 0.05), whereas there was no different concentration of cer (d18:1/18:0) observed between groups. **b.** concentration of cer (d18:1/24:1) and cer (d18:1/24:0): there was no difference in cer (d18:1/24:1) concentration between groups, whereas cer (d18:1/24:0) concentration was significant increased in AMI compared to it in UAP and no difference compared to it in atherosclerosis(**P < 0.01). **c.** ratio of ceramides: there was only a significant increased ratio of cer (d18:1/24:1)/cer (d18:1/24:0) in AMI compared to it in UAP (*P* < 0.05). (data is presented as mean ± SEM)

The patients were assigned into 4 groups according to coronary artery stenosis (stenosis < 25%, 25–50%, 50–75 and > 75%). Kruskal-Wallis test was used to analyze and compare the ceramide levels in group 1, 2, 3 and 4. There was no significant difference in levels of Cer (d18:1/16:0), Cer (d18:1/18:0), Cer (d18:1/24:1), or ratios of Cer (d18:1/16:0), Cer (d18:1/18:0) or Cer (d18:1/24:1) to Cer (d18:1/24:0) between groups, except Cer (d18:1/16:0)/Cer (d18:1/24:0) ratio was significantly increased in group 1 compared to group 2 (*P* < 0.05, Fig. 3). Moreover, plasma ceramides levels were analyzed regarding different types of coronary artery diseases. It was found that 78.8% of patients admitted to hospital were diagnosed as UAP, 10.7 and 6.9% were diagnosed as AMI and atherosclerosis, respectively. The remaining cases included rheumatic heart disease, sinus tachycardia, pre-excitation syndrome and other heart diseases. The levels of plasma Cer (d18:1/16:0), Cer (d18:1/24:0), and Cer (d18:1/24:1)/ Cer (d18:1/24:0) ratio were significantly increased in AMI patients compared to those in UAP patient (Fig. 4), whereas there was no difference in ceramide levels or ratios between other groups. It is worthy to point that the incidence rates of established diseases were different in 4 groups (data not shown), which suggested that difference of established disease is a risk factor in coronary artery stenosis.

**Fig. 5:**
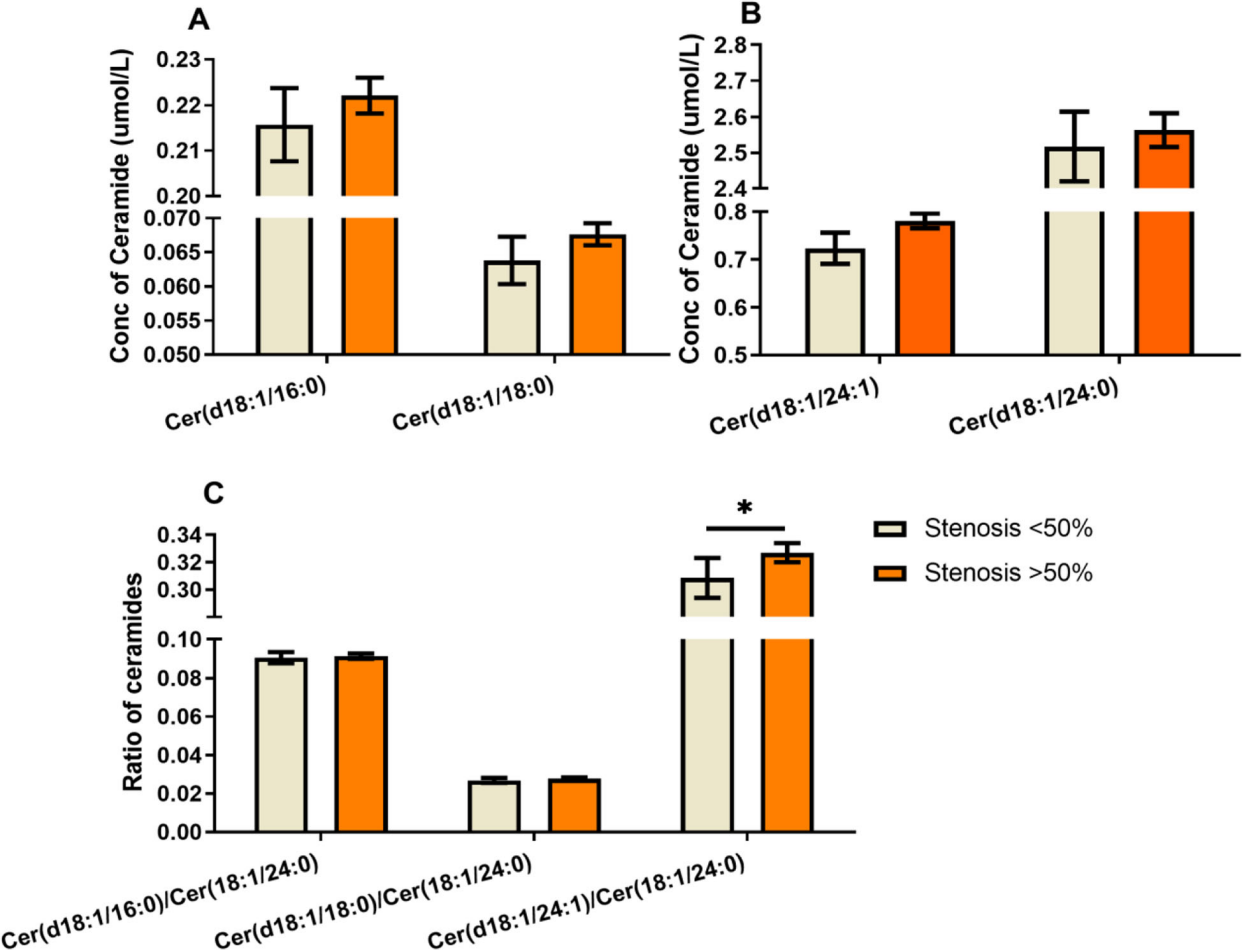
Concentration of ceramides and ratio of ceramides to cer (d18:1/24:0): There was a significant increased ratio of cer (d18:1/24:1)/cer (d18:1/24:0) in group of stenosis > 50% compared to group of stenosis < 50% (*P <* 0.05), whereas no difference was observed in other ceramides and ratios of ceramides between groups. (data is presented as mean ± SEM)

Although no significant increase of plasma levels of ceramides were observed with severity of coronary artery stenosis, the incidences of UAP, AMI and atherosclerosis were different in subjects with different severity of stenosis. Those results suggested that the association between coronary artery stenosis and ceramide level was possibly adjusted by the diagnosis during index hospitalization. In addition, the average age of cohort was more than 55 years old and there were age-related disease conditions and life styles such as smoking habbit that could influence atherosclerosis besides ceramides. Thus, multiple logistic stepwise regression analysis was used to evaluate the independent association between plasma ceramide and severity of atherosclerosis after adjustment for established risk factors. The model was adjusted for age, gender, smoking, pre-existing myocardial/cerebral infarction, the presence of hypertension, lipids, hemoglobin A1c% (HbA1c%) and the diagnosis during index hospitalization. Covariates included in multiple regression model were selected as potential confounding factor based on their significance in univariate analyses or their biological plausibility. To enhance the power of the logistic stepwise regression analysis, the subjects were re-assigned into 2 groups in order to increase the number of sample in each group: group A with mild to moderate stenosis (group1 + group2, i.e. coronary stenosis < 50%) and group B with moderate to severe stenosis (group3 + group4, i.e. coronary stenosis > 50%). As shown in Fig. 5, only Cer (d18:1/24:1)/Cer (d18:1/24:0) ratio was significantly increased in group B compared to group A (*P* < 0.05) before logistic stepwise regression analysis.

Logistic stepwise regression analysis was performed to analyze the effect of multiple factors on coronary artery stenosis with entry and exit criteria of 0.2 for stepwise selection. In model 1, the results show that high level of Cer (d18:1/24:1)/Cer (d18:1/24:0) (odds ratio (OR) 18.927, 95% confidence interval (CI) 1.902–188.380), female gender (OR 2.57, 95% CI 1.446–4.567), high level of HbA1c% (OR 1.37, 95% CI 1.012–1.854) and AMI compared with atherosclerosis (OR 436.227, 95% CI 45.285- > 999.999) were associated with more severe coronary artery stenosis (Table 2). In model 2, in addition to female gender (OR 2.609, 95% CI 1.401–4.859), high level of Cer (d18:1/24:1)/Cer (d18:1/24:0) (OR 25.152, 95% CI 2.360–268.084) and AMI compared with atherosclerosis (OR 436.9.8, 95% CI 44.311- > 999.999) were associated with more severe coronary artery stenosis, low level of LDL-C (OR 1.579, 95% CI 1.046–2.384) and UAP compared with atherosclerosis (OR 70.024, 95% CI 21.017–233.300) were also associated with more severe coronary artery stenosis (Table 2).

**Table 2:** Association between plasma ceramides and the severity of coronary stenosis after adjustment of multiple risk factors

Logistic Stepwise Regression Analyses	Effect	β	***P***	Odds Ratio	95% Confidence Interval
**Model 1**^**a**^	Gender (male vs female)	−0.472	0.001	2.57	1.446–4.567
	HbA1c%	0.315	0.042	1.37	1.012–1.854
	Cer (d18:0/24:1)/Cer (d18:0/24:0)	2.941	0.012	18.927	1.902–188.380
	Diagnosis UAP vs atherosclerosis	0.733	0.069	62.903	19.586–202.016
	Diagnosis AMI vs atherosclerosis	2.676	< 0.001	439.227	45.285- > 999.999
**Model 2**^**a**^	Gender (male vs female)	0.480	0.003	2.609	1.401–4.859
	Hypertension (yes vs. no)	−0.235	0.121	0.625	0.346–1.131
	HDL cholesterol	−0.888	0.098	0.412	0.144–1.177
	LDL cholesterol	0.467	0.03	1.579	1.046–2.384
	HbA1c%	0.242	0.129	1.273	0.932–1.739
	Cer (d18:0/24:1)/Cer (d18:0/24:0)	3.225	0.008	25.152	2.360–268.084
	Diagnosis UAP vs atherosclerosis	0.806	0.049	70.024	21.017–233.300
	Diagnosis AMI vs atherosclerosis	2.637	< 0.001	436.9.8	44.311- > 999.999

^a^adjustment factor.model 1: gender, age, smoking, prior MI/CI, current diagnosis, HbA1c%. model 2: gender, age, smoking, prior MI/CI, the presence of hypertension, current diagnosis, HbA1c%, TC, HDL-C, LDL-C. entry and exit criteria were 0.2 for stepwise selection

### MACE and the prognostic model

**Fig. 6:**
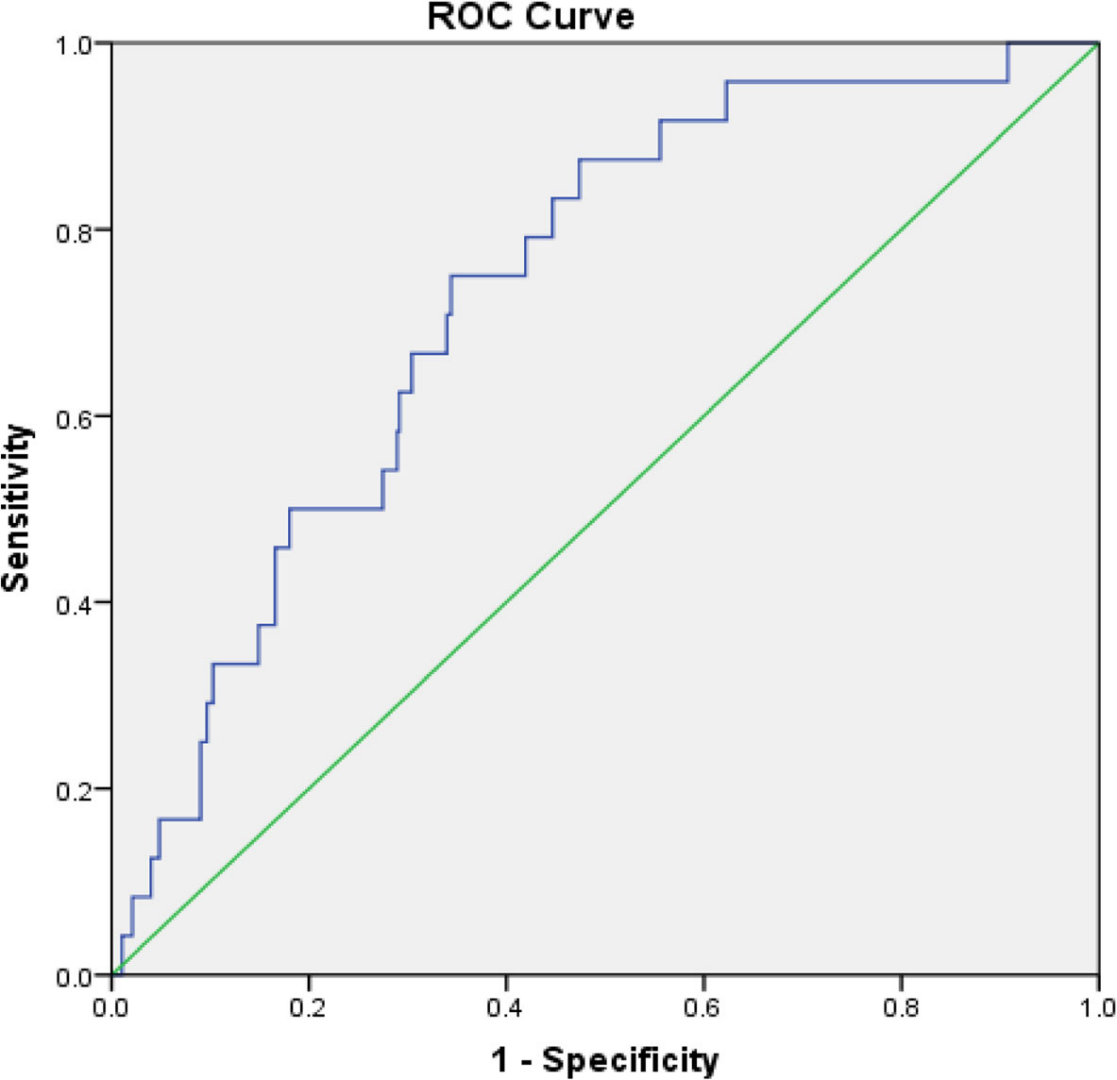
The ROC curve of prognostic model for prediction of MACE. AUC of ROC was 0.732 and 95% CI was 0.642–0.822

A total of 553 subjects were followed up for 1 year after angiography. The one-year incidence of MACE was 6.01% (6.32% in male and 5.24% in female), and the one-year mortality was 0.45%. The prognostic association of individual ceramide species with MACE was modelled using multiple logistic regression for binary outcomes (event, non-event). After clinical risk factors adjustment including concentrations of targeted ceramides, stenosis severity, infarction history, diagnosis during index hospitalization, gender, age, smoking, and HbAc1% inmultiple logistic regression model, the results showed that pre-existing infarction history was associated with MACE (OR 2.874, 95% CI 1.139–7.254, *P* = 0.025), whereas stenosis severity (OR 6.732, 95% CI 0.730–62.054, *P* = 0.092) and other factors were not. Receiver operating characteristics (ROC) for the prognostic model was shown in Fig. 6**.** The area under curve (AUC) was 0.732 and 95% CI was 0.642–0.822, which indicated that the established prognostic model gave a good discrimination.

## Discussion

The present study investigated three of the most reported ceramides in ACS studies, Cer (d18:1/16:0), Cer (d18:1/18:0) and Cer (d18:1/24:1), and their plasma ratios to Cer (d18:1/24:0) in patients with different severity of coronary stenosis. It was firstly found that the ratio of Cer (d18:1/24:1) to Cer (d18:1/24:0) was significantly increased in subjects with stenosis > 50% compared to those with stenosis < 50% before logistic regression analysis. Moreover, the higher levels of Cer (d18:1/16:0), Cer (d18:1/24:0) and the higher ratio of Cer (d18:1/24:1) to Cer (d18:1/24:0) were observed in AMI patients compared to UAP patients. These results are not consistent with the findings reported in a recent study [20], which investigated 12 ceramides species both in AMI patient and rat AMI model. This study found that only plasma Cer (d18:1/24:0) level was significantly increased in AMI subjects compared with sham and healthy control subjects, whereas no significant changes in other ceramide levels including Cer (d18:1/16:0), Cer (d18:1/18:0) and Cer (d18:1/24:1) was observed. However, it is worthy to point out that the present study only found certain ceramide differences between AMI patients and other cardial artery diseases pateints but not between AMI patients and the healthy control. A study in myocardial perfusion defects [14] found that only Cer (d18:1/24:1) was significantly increased in subjects with myocardial perfusion defects compared with those without myocardial perfusion defects in unadjusted model, whereas most targeted ceramides (Cer (d18:1/16:0), Cer (d18:1/18:0), Cer (d18:1/20:0), Cer (d18:1/22:0) and Cer (d18:1/24:1)) were associated with myocardial perfusion defects after adjustment for cardiovascular risk factors. In addition, the accumulating experimental and clinical evidences indicate that there is a potential role of distinct ceramide species in atherosclerotic plaque progression, vulnerability and ischemia-induced cardiomyocyte apoptosis. For example, the distinct plasma ceramides that are involved in endothelial damage and atherosclerotic plaque erosion/vulnerability can further induce thrombosis and AMI [9, 11, 13, 21–26]. All these evidences suggest that the specific ceramide species and ratio of ceramides are likely to be specifically involved in a certain type of myocardial ischemia or insufficient blood supply. Therefore, it is hypothesized that ceramides may facilitate the accurate diagnosis AMI, UAP and other coronary artery diseases. In the present study, it was observed that 56% of mild coronary artery stenosis (< 50%) were diagnosed as ACS (AMI: 1%; UAP: 55%). The pathophysiologic background about the mild coronary artery stenosis evokes ACS is not clear yet. A recent review [27] reported that the prevalence of myocardial infarction with normal coronary arteries (MINCA) and myocardial infarction in the absence of obstructive coronary artery disease (stenosis > 50%) are 1–15% of all myocardial infarctions. And those MINCA patients showed lower prevalence of dyslipidemia, hypertension, diabetes and other traditional risk factors for CAD. In this review, the authors summarized the possible mechanisms of MINCA including concealed atherosclerosis, inflammation, arteritis, catecholamine-induced cardiomyopathy and myocarditis.

Although the present study found that ratio of Cer (d18:1/24:1) to Cer (d18:1/24:0) was significantly higher in patients with stenosis > 50% compared with those in patients with stenosis < 50% before adjustment, it is common that patients with coronary artery stenosis suffer from other metabolic and cardiovascular diseases. In addition, ceramides have been proposed as novel biomarkers for several diseases [28] such as cancer, diabetes, Alzheimer’s disease, depression, multiple sclerosis, as well as coronary artery disease. Therefore, multiple risk factors including age, gender, pre-existing myocardial/cerebral infarction, HbA1c%, smoking, lipids, the presence of hypertension and the diagnosis during index hospitalization were adjusted in multiple logistic stepwise regression analysis and the results indicated that a high level of Cer (d18:0/24:1)/Cer (d18:0/24:0), high level of HbA1c%, female gender and AMI (compared with atherosclerosis) are significantly associated with more severe coronary artery stenosis. For the first time that the association between high level of Cer (d18:0/24:1)/ Cer (d18:0/24:0) ratio and more severe coronary artery stenosis is reported, and there was no association between any specific ceramide and severity of coronary artery stenosis found in the present study. Recently, Mantovani and colleagues [29] reported that a higher level of plasma Cer (d18:1/24:0), but not Cer (d18:0/24:1), was associated with a greater severity of LAD stenosis after adjustment for cardiovascular risk factors. However, Cer (d18:0/24:1)/Cer (d18:0/24:0) ratio was not investigated in this study. In addition, it is noteworthy that the patients underwent elective and urgent angiography were enrolled in Mantovani’s study, whereas patients who needed urgent angiography were excluded in present study. Therefore, ratio of AMI patients to all subjects in Mantovani’s study was possibly different from it in present study. As aforementioned, ceramides levels were higher in AMI than other CAD. Moreover, levels of plasma ceramides were higher in AMI patients with plaque rupture than those with plaque erosion [30]. It is suggested that AMI and plaque status underlying CAD correlate with levels of plasma ceramides and current diagnosis should be considered as covariate adjusted in logistic regression analysis.

The observed association between female gender and severe coronary artery stenosis was possibly resulted from estradiol-induced unbalance of ceramides. A recent study [31] using multivariable linear regression analysis revealed that Cer (d18:1/24:0) and Cer (d18:1/24:1) were increased over age in women. In women of all ages, but not in men, plasma Cer (d18:1/24:1) was negatively correlated with plasma estradiol. In addition, this study found that estradiol significantly decreased ceramide accumulation in in-vitro study, which supported the relationship between estradiol and ceramide.

The present study found that incidence of AMI had a more significant association with severity of coronary artery stenosis than atherosclerosis. As discussed above, some studies suggested that plasma ceramides regulate atherosclerotic plaque erosion, which may possibly result in AMI [9, 11, 13, 23, 26]. In addition, arecent study [20] reportedthat a higher concentration of plasma ceramides from patients with recent AMI compared with those without recent AMI. Consistently, the present study showed that the plasma levels of Cer (d18:1/16:0), Cer (d18:1/18:0), Cer (d18:1/24:1), Cer (d18:1/16:0)/ Cer (d18:1/24:0) and Cer (d18:1/24:1)/Cer (d18:1/24:0) were significantly increased in AMI patients compared with UAP patients without adjusting risk factors. However, no significant difference in levels of plasma ceramides between AMI and atherosclerosis patients was observed without risk factor adjustment. Meanwhile, it is also worthy to point out that there was only 68 out of 69 AMI patients recruited in group with stenosis > 50% in present study, whereas 6 out of 38 atherosclerosis patients recruited in group with stenosis > 50%. Therefore, an indifferent ceramide level observed in present study may result from the small size of subjects.

The present study also investigated MACE in 1 year after index hospitalization. After adjusting concentrations of targeted ceramides and other risk factors in multiple logistic regression model, the present study found that pre-existing infarction was associated with MACE, whereas none of ceramide showed an independent association with MACE. This is not consistent with some recent studies [9–11, 32], which showed that specific plasma ceramides including Cer (d18:1/16:0), Cer (d18:1/18:0) and Cer (d18:1/24:1) predicted the development of MACE both in patients with ACS and healthy subjects, independently of cardiovascular risk factors. It is important to note that these findings were based on a long-term MACE investigation, which included a median follow-up of 4.6 years investigation in Laaksonen’s study. In addition, a study [12] reported that the risk of developing MACE in CAD patients with elevated levels of distinct plasma ceramides was potentially reduced by Mediterranean diet. Therefore, the dietary, medication and other factors should be investigated in the further study of MACE.

### Study strengths and limitations

The present study was the first work to report the association between plasma ceramide level and these verity of coronary stenosisin patients with ACS. However, no association was established between plasma ceramide level and MACE. The limitation of the present study is the 1 year of follow-up MACE investigation period, which is probably inadequate to study the long-term effect of ceramide in CAD patients.

## Conclusion

Three ceramides (Cer (d18:1/16:0), Cer (d18:1/18:0), Cer (d18:1/24:1)) and their ratios to Cer (d18:1/24:1) are mostly investigated in the pathologenesis study of ACS. These ceramides have been proposed to be used in clinical practice as ACS biomarkers. However, few study investigated these ceramide levels in patients regarding different severity of coronary stenosis and clinical diagnosis (i.e. AMI, UAP and astherosclerosis). The present study reported that Cer (d18:1/16:0), Cer (d18:1/24:0) and ratio of Cer (d18:1/24:1) to Cer (d18:1/24:0) were associated with the development of stenosis. Moreover, the association between Cer (18:0/24:1)/Cer (18:0/24:0) and severity of coronary artery stenosis with ACS, but not with other CAD were observed for the first time. These results indicated that plasma ceramides levels can be used to estimate the severity of stenosis and facilitate the diagnosis of ACS in clinical practice. However, this study did not establish the predictive effect of ceramide on MACE. Although a ceramide plasma test for the prediction of cardiac risk was available commercially since 2016 by the Mayo Medical Laboratories [33], the species of ceramides of interests vary from different ACS to coronary artery disease and myocardial ischemia/perfusion defects. Further study is needed to investigate the level of specific and/or de novo ceramides in pathogenesis of different ACS and other coronary artery diseases, as well as a long-term MACE investigation plan.

## Supplementary information

**Additional file 1.**

## Data Availability

The datasets used or analysed during the current study are available from the corresponding author on reasonable request.

## Abbreviations

ACS: Acute coronary syndrome
AMI: Acute myocardial infarction
AUC: Area under curve
BNP: Brain natriuretic peptide
CABG: Coronary artery bypass grafting
CAD: Coronary artery disease
Cer: Ceramide
CI: Confidence interval
CK-MB: Creatine kinase-MB
cTnl: cardiac troponin I
ECG: Electrocardiograph
HbA1c%: Hemoglobin A1c%
HDL: High-density lipoprotein
hs-CRP: high-sensitivity C-reactive protein
IS: Internal standard
LC-MS/MS: Liquid chromatography-tandem mass spectrometry
LDL: Low density lipoprotein
MACE: Major adverse cardiavascular and cerebrovascular
NSTEMI: Non-ST-segment elevation myocardial infarction
OR: Odds ratio
PCI: Percutaneous coronary intervention
ROC: Receiver operating characteristics
STEMI: ST-segment elevation myocardial infarction
TC: Total cholesterol
UAP: Unstable angina pectoris
