# Supplementary material for: Association between ceramides and coronary artery stenosis in patients with coronary artery disease
Source: Lipids Health Dis. 2020 Jun 25;19:151. doi: 10.1186/s12944-020-01329-0 (PMC7315545; doi:10.1186/s12944-020-01329-0)
Supplement: Supplementary file 1 — Additional file 1. [file 12944_2020_1329_MOESM1_ESM.docx]

**Method of Ceramide Measurement**

**Chemicals and reagents**

N-Palmitoyl-D-erythro-sphingosine/Cer(d18:1/16:0), Nstearoyl-D-erythro-

1. sphingosine/Cer(d18:1/18:0), Nlignoceroyl-D-erythro-sphingosine/Cer(d18:1/24:0), Nnervonoyl-D-erythro-sphingosine/Cer(d18:1/24:1) standards, as well as their isotopic-labeled ones, D7-Cer(d18:1/16:0), D7-Cer(d18:1/18:0), D7-Cer(d18:1/24:0) and D7-Cer(d18:1/24:1) were purchased from Beijing Health Biotech Co. Ltd., Beijing, China. Methanol, acetonitrile and 2-propanol (HPLC grade) were purchased from Fisher Scientific (Waltham, Massachusetts, USA). 5% bovine serum albumin (BSA) was purchased from Sigma-Aldrich (Louis, Missouri, USA). Ethyl acetate and ammonium acetate were purchased from Alfa Aser (Heysham, Lancashire, UK).

**Sample collection**

Blood samples were centrifuged (1000-1200 g, 15 min) within 2 hours after collection, and the upper plasma (>500 μL) was carefully transferred into an EP tube. The plasma samples can be stored at room temperature for up to 8 hours during sample pretreatment process. Otherwise, samples should be transferred to a 2-8 ℃ refrigerator for short-term storage for up to 48 hours, or under -20 ℃ for long-term storage up to 3 months.

**Optimization of sample** **pretreatment**

To achieve the optimal recovery of ceramides from plasma matrices, the choice of protein precipitant reagent was of essential importance to the success of the whole assay. Thus, several precipitant reagents, *i.e.* methanol, acetonitrile, isopropanol, and ethyl acetate/isopropanol (1:4) were investigated during the sample pretreatment study. After preparation, samples were subjected to LC-MS/MS assayand the extraction rates of different precipitants were compared, *i.e.* response ceramides, matric interferences. Generally, isopropanol and ethyl acetate/isopropanol (1:4) showed equivalent efficiency, which were much higher than methanol and acetonitrile. Finally, isopropanol was selected as the sample precipitation reagent. In addition, isopropanol was found to have significant positive effect on the stability of ceramidesin plasma samples.

The effects of sample and reagent volume employed during the precipitation of plasma were also investigated. It was shown that the volume of isopropanol had a significant impact on the ceramide extraction efficiency. Therefore, a trial was conducted in order to clarify the effect of precipitant, involving volume ratios 1:15, 1:45, 1:60, 1:75 of plasma to isopropanol. In the end, a ratio of plasma/isopropanol of 1:60 was adopted in the whole pretreatment.

A comparison experiment of incubation time before centrifugation (1, 5, 10 min) was also carried out. The results showed that different extraction time had similar effect on the recovery ratios of ceramides in plasma. Therefore, the incubation time can be limited to 1 min for the convenience of practical operation.

**Sample and calibrator pretreatment procedure**

Individual standard stock solutions (1 mg/mL) were prepared in 2-propanol. All stock solutions were stored at -80 ^o^C until experiments. Working solutions for calibration containing Cer(d18:1/16:0), Cer(d18:1/18:0), Cer(d18:1/24:0), and Cer(d18:1/24:1) were prepared by serial dilutions freshly. Internal standard (IS) solution containing D7-Cer(d18:1/16:0) (0.05 μg/mL), D7-Cer(d18:1/18:0) (0.025 μg/mL), D7-Cer(d18:1/24:0) (0.5 μg/mL), and D7-Cer(d18:1/24:1) (0.5 μg/mL) was added to each calibrator. The linear ranges of ceramides were 0.005 μg/mL~1 μg/mL for Cer(d18:1/16:0) and Cer(d18:1/18:0), 0.05 μg/mL~10 μg/mL for Cer(d18:1/24:0) and Cer(d18:1/24:1), which also covered the upper and lower detection limits for the 4 ceramides.

Plasma samples were thawed at 4 ^o^C or room temperature successively before pretreatment, and repeated freeze-thaw cycles should be avoided. An aliquot of 10 μL plasma was transferred into a 96-well plate. 20 μL of internal standards working solution was added followed by 570 μL of isopropanol for protein precipitation. The final mixture was vortexed for 3min at room temperature, and centrifuged at 1000 g for 15 min at 4 ^o^C. The supernatant was transferred into another 96-well sample collecting plate waiting for LC-MS/MS analysis.

**Instrumentation**

Ceramides were analyzed using the Shimadzu LC-20A system, and an AB SCIEX triple Quad 3200 tandem mass spectrometer with an electrospray ion source (ESI). The mass spectrometer was operated in positive ion mode and the quantification was achieved in MRM mode. An BEH C18 column (1.7 μm 2.1 × 50mm id.) from Waters (Milford, MA) was used in analytes separation. Mobile phase A were prepared using 0.1% formic acid in 0.01 mol/L ammonium acetate-water and B were prepared using 0.1% formic acid-acetonitrile. A gradient of 0 min, 15% A; 1 min, 0% A; 4 min, 0% A; 5.4 min, 15% A; 5.5 min, 15% A with a flow rate of 0.5 mL/min was used. The column temperature was kept at 45 ℃ throughout the analysis. The injection volume of standard and samples was 5 μL. Detail information of fragment ion for each analyte was shown in **Table S1**.

| **Table S1 MRM parameters for ceramides** | | | | | | |
| --- | --- | --- | --- | --- | --- | --- |
| Analyte | Parent ions | Daughter ions | DP | CE | CXP | Internal standard |
|  | m/z | m/z |  |  |  |  |
| 16:00 | 538.5 | 264.4 | 40 | 35 | 4 | D7-16:0 |
| 18:00 | 566.4 | 264.4 | 41 | 41 | 4 | D7-18:0 |
| 24:00 | 650.5 | 264.4 | 47 | 41 | 3 | D7-24:0 |
| 24:01 | 648.4 | 264.4 | 38 | 49 | 4 | D7-24:1 |
| D7-16:0 | 545.2 | 271.4 | 59 | 39 | 4 | — |
| D7-18:0 | 573.5 | 271.4 | 62 | 35 | 4 | — |
| D7-24:0 | 657.5 | 271.5 | 74 | 44 | 4 | — |
| D7-24:1 | 655.5 | 271.4 | 47 | 49 | 4 | — |

**Validation of the method**

We evaluated the method performance according to CLSI C62-A, liquid chromatography-mass spectrometry methods, approved guideline.

**Statistical methods**

The comparison of normal distribution data was conducted by t-test, and the comparison of non-normal distribution data was conducted by Mann-Whitney U test, Chi square test was used to compare the composition ratio of enumeration data between groups.

*P*<0.05 was considered statistically significant. SPSS version 25.0 was used for statics analysis.

**Calibration and sensitivity**

The proposed method was evaluated by its linearity and sensitivity. Standard calibration was performed periodically during the [method](http://www.baidu.com/link?url=JX80OlObZAxJvQrlTLviyL3lRbbHhje43znmML4e6AJQoeIFV9fX-l3MJ0qmA2WPgZ_jdf0aIHeMbPk2vNOweSGxcJNevn65pN4XE2Cjj0a) investigation and sample analysis. Generally, the calibration linearity of ceramides was performed by plotting the ratio of peak response of ceramides to the peak response of their own IS stable isotope in working standard solutions versus the quantity of ceramides. Three levels of standard solutions, QCL, QCM and QCH, were employed as the quality control, and was checked every 10 injections to ensure the stability of analysis and calibration verification. All the ceramides showed fine linearity within the calibration range, with correlation coefficient (R^2^) better than 0.99 (**Fig.S1**). Limit of detection (LOD) was also determined by diluting of the lowest level calibration standard solution, with signal-to-noise (S/N) ratios better than 3. Both plasma-and reagent-based calibration were investigated during the analysis, in order to evaluate the matrix infection. When comparing the linearity of plasma calibration curve with that of isopropanol calibration curve, it showed that two different matrices have similar linearity and correlation coefficient in a given linear range. It was confirmed that isopropanol matrix can replace plasma matrix as a calibration curve matrix for various ceramide detection.

**Quality control and Method performance**

Intra-batch and inter-batch precision of the method was determined. A selected plasma sample was employed as the quality control. Generally, for intra-batch precision, 10 replicates of the QCL, QCM, QCH standard solutions and the quality control plasma were prepared parallelly and injected on the same day. For intra-batch precision study, 3 batches of ceramides kits were analyzed following the same procedure of intra-batch precision. The concentrations of Cer(d18:1/16:0)and Cer(d18:1/18:0) in QCL, QCM, QCH standard solutions were 0.01, 0.1, 0.75 μg/mL, and the concentrations of Cer(d18:1/24:0) and Cer(d18:1/24:1) in QCL, QCM, QCH standard solutions were 0.1, 1.0, 7.5 μg/mL, respectively (**Fig.S2**). The intra-batch and inter-batch relative standard deviations (RSDs) ranged from 4.7%~11.6%, which were detailed in **Table S2**.

| **Table S2 Spiked recoveries of ceramides in human plasma (n=3)** | | | | | | | | | | | | |
| --- | --- | --- | --- | --- | --- | --- | --- | --- | --- | --- | --- | --- |
|  | Batch1 | | | | Batch 2 | | | | Batch 3 | | | |
| Analytes | Plasma 1 | Plasma 2 | Plasma 3 | Plasma 4 | Plasma 1 | Plasma 2 | Plasma 3 | Plasma 4 | Plasma 1 | Plasma 2 | Plasma 3 | Plasma 4 |
| Cer(d18:1/16:0) | 110 | 89.3 | 99.1 | 95.2 | 113.3 | 91 | 101.4 | 97.4 | 113.3 | 93 | 103.5 | 99.3 |
| Cer(d18:1/18:0) | 110.3 | 95.2 | 100.2 | 95.2 | 106 | 90.9 | 95.8 | 90.9 | 108.7 | 93 | 98.2 | 93.3 |
| Cer(d18:1/24:0) | 90 | 94 | 93.7 | 88.5 | 90 | 92.3 | 92.3 | 87.1 | 93.3 | 92.3 | 92.5 | 87.4 |
| Cer(d18:1/24:1) | 111.3 | 99.4 | 97.3 | 94 | 110 | 97.7 | 96.2 | 93.1 | 109.7 | 97.1 | 95.6 | 92.5 |

**Accuracy and Specificity**

The accuracy was evaluated by spiked recovery. Recoveries were calculated by adding a certain volume of standard solution to the human plasma samples, and each spiked concentration were analyzed for three replicates. Three batches of ceramide determination calibration/kits were involved. For the possible endogenous ceramides, the background value needed to be deducted during the recovery calculation. According to the data in **Table S3**, the spiked recoveries of ceramidesranged 87.1%~111.3%.

| **Table S3 Stability of plasma at pre- and post-pretreatment** | | | | | |
| --- | --- | --- | --- | --- | --- |
| Analytes | Time | Plasma (CV%) | | Post pretreatment (CV%) | |
|  |  | Ambient | 2-8 ℃ | Ambient | Autosampler |
|  |  | Temperature |  | Temperature |  |
| Cer(d18:1/16:0) | 0 h | -3.4 | 5.7 | 2.5 | -11.3 |
|  | 4 h | -6.7 | -3.6 | -3.3 | 5.6 |
|  | 8 h | 5.1 | -7.9 | 7.9 | 6.2 |
|  | 12 h | -1.7 | 3.2 | -1.9 | 5.3 |
|  | 24 h | 3.5 | -5.7 | 2.2 | -5.5 |
|  | 48h | -5.9 | 4.3 | 2.3 | 7.2 |
| Cer(d18:1/18:0) | 0 h | -1.7 | 10.2 | 7.5 | -11.7 |
|  | 4 h | -1 | -1.6 | -3.4 | 7.2 |
|  | 8 h | 7.7 | 8.8 | 3.5 | -1 |
|  | 12 h | -7.2 | 0.8 | 0.1 | -9.9 |
|  | 24 h | -13.9 | 1.8 | 4.6 | -6.6 |
|  | 48h | -2.21 | -4.5 | 3.1 | 7.8 |
| Cer(d18:1/24:0) | 0 h | 1.5 | 5.1 | -6.6 | -11.4 |
|  | 4 h | -5.8 | 0.6 | -2.4 | -3.8 |
|  | 8 h | -6.5 | 6.8 | -8.2 | -7.5 |
|  | 12 h | 1.5 | -12.9 | -7.8 | -10.1 |
|  | 24 h | -7.9 | 8.8 | -3 | -14.3 |

Specificity was evaluated by interference effects of interferences of high concentration of interferences deliberately added to plasma. Generally, the possible interfering substances, hemoglobin (2 g/L), bilirubin (342 μmol/L) and triglyceride (37 mmol/L) were studied respectively. After routine pretreatment procedure, ceramides spiked into the plasma samples containing spiked interferences were evaluated by their chromatographic behavior, as well as spiked recoveries (n=6). It was shown that ceramides in plasma in the presence of high concentrations of interfering substances were consistent with those in plasma without interfering substances, indicating that the method’s high specificity.

**Stability**

Stability studies included stability studies of ceramide determination kits (standard solutions) and plasma samples.

The stability ceramide standard solutions at room temperature, 2~8 ℃ storage, as well as stability during repeated freezing and thawing, has been studied respectively. The results proved that the standard solutions were stayed stable for up to 4 weeks when placed under ambient temperature. When stored under 2~8 ℃ and -20 ℃, the expiration date could be prolonged to 6 months, even under frequent freezing and thawing.

The stability evaluation of ceramidein plasma during analysis mainly includes the following two aspects: (1) the stability in plasma, such as short-term stability at room temperature, as well as transportation (2-8 ℃); (2) post treatment stability, including stability of post-treatment placement at room temperature and auto-sampler. Ceramide was proved to be stable in plasma as well as in pretreated plasma samples (**Table S4**).

| **Table S4 MRM parameters for ceramides** | | | | | | |
| --- | --- | --- | --- | --- | --- | --- |
| Analyte | Parent ions | Daughter ions | DP | CE | CXP | Internal standard |
|  | m/z | m/z |  |  |  |  |
| 16:00 | 538.5 | 264.4 | 40 | 35 | 4 | D7-16:0 |
| 18:00 | 566.4 | 264.4 | 41 | 41 | 4 | D7-16:0 |
| 24:00:00 | 650.5 | 264.4 | 47 | 41 | 3 | D7-16:0 |
| 24:01:00 | 648.4 | 264.4 | 38 | 49 | 4 | D7-24:1 |
| D7-16:0 | 545.2 | 271.4 | 59 | 39 | 4 | — |
| D7-18:0 | 573.5 | 271.4 | 62 | 35 | 4 | — |
| D7-24:0 | 657.5 | 271.5 | 74 | 44 | 4 | — |
| D7-24:1 | 655.5 | 271.4 | 47 | 49 | 4 | — |
